# Maternal high fat diets: impacts on offspring obesity and epigenetic hypothalamic programming

**DOI:** 10.3389/fgene.2023.1158089

**Published:** 2023-05-11

**Authors:** Begüm Harmancıoğlu, Seray Kabaran

**Affiliations:** Department of Nutrition and Dietetics, Faculty of Health Sciences, Eastern Mediterranean University, Famagusta, Türkiye

**Keywords:** maternal obesity, maternal overnutiriton, high fat diet, hypothalamic programming, fetal programming

## Abstract

Maternal high-fat diet (HFD) during pregnancy is associated with rapid weight gain and fetal fat mass increase at an early stage. Also, HFD during pregnancy can cause the activation of proinflammatory cytokines. Maternal insulin resistance and inflammation lead to increased adipose tissue lipolysis, and also increased free fatty acid (FFA) intake during pregnancy (˃35% of energy from fat) cause a significant increase in FFA levels in the fetus. However, both maternal insulin resistance and HFD have detrimental effects on adiposity in early life. As a result of these metabolic alterations, excess fetal lipid exposure may affect fetal growth and development. On the other hand, increase in blood lipids and inflammation can adversely affect the development of the liver, adipose tissue, brain, skeletal muscle, and pancreas in the fetus, increasing the risk for metabolic disorders. In addition, maternal HFD is associated with changes in the hypothalamic regulation of body weight and energy homeostasis by altering the expression of the leptin receptor, POMC, and neuropeptide Y in the offspring, as well as altering methylation and gene expression of dopamine and opioid-related genes which cause changes in eating behavior. All these maternal metabolic and epigenetic changes may contribute to the childhood obesity epidemic through fetal metabolic programming. Dietary interventions, such as limiting dietary fat intake <35% with appropriate fatty acid intake during the gestation period are the most effective type of intervention to improve the maternal metabolic environment during pregnancy. Appropriate nutritional intake during pregnancy should be the principal goal in reducing the risks of obesity and metabolic disorders.

## Introduction

Pregnancy is a critical period for optimal fetal growth and development. The diet which is consumed by the mother during pregnancy can influence the health of both the mother and the child (Khaire et al., 2020). For many years, it has been known that some specific environmental factors throughout gestation (e.g., maternal nutrition, maternal lifestyle, metabolic diseases) may cause changes in gene expression and permanently damage the structure and function of some specific organs of the fetus. Therefore, many non-communicable diseases such as obesity, cardiovascular disease, diabetes, hypertension, kidney disease, allergic disease, nonalcoholic fatty liver disease, neurocognitive impairments, and metabolic syndrome may occur later in life (Mennitti et al., 2015; Hsu and Tain, 2019). Besides maternal nutrition, adverse maternal physiology is also associated with poor health of the offspring and later development of chronic diseases (Shrestha et al., 2020). This phenomenon was first reported as Barker’s Hypothesis by British epidemiologist David Barker in 1993, and it was also called as “fetal origins of adult disease” and “developmental origins of health and disease”. As of 2002, Barker’s Hypothesis is generally known as the “Developmental Origins of Health and Disease (DOHaD)” and is currently referred to as fetal programming (Padmanabhan et al., 2016; Khaire et al., 2020; Shrestha et al., 2020).

Obesity has become a major global health problem because of health-related risk factors. It is well known that non-communicable diseases compose the main cause of death worldwide. Increased consumption of diets rich in calories and specific macronutrients such as carbohydrates and fats have raised interest in the underlying mechanisms of development of obesity (Gawlińska et al., 2020). Previous evidence shows that the prevalence of overweight and obesity among children, adolescents, adults, and also women of childbearing age has been increasing over the past years (Ng et al., 2014; Padmanabhan et al., 2016). Considering this increasing, it has been demonstrated that maternal obesity and maternal HFD (˃35% calories from fat) intake have been associated with adverse perinatal outcomes, childhood obesity and obesity-related poor health outcomes later in life (Freeman, 2010; Ota et al., 2011; Gaillard et al., 2013; Rincel et al., 2016). Additionally, current literature strongly indicates that maternal HFD and excessive weight gain during both pregnancy and lactation alter DNA methylation and gene expression of hypothalamic appetite-related neurons and central reward system molecules, which cause changes in appetite and eating behavior in offspring (Vucetic et al., 2010; Desai et al., 2016).

In this review, we discuss the effects of maternal excess weight gain and maternal obesity on fetal programming of obesity. Besides, the impacts of maternal HFD on fetal programming of obesity and their potential role in epigenetic hypothalamic programming are also discussed throughout the manuscript. Finally, we summarize the evidence for the long-term effects of maternal HFDs on the growing burden of childhood obesity.

### Mechanisms of developmental programming of maternal obesity and prenatal programming

The term “developmental programming” refers to a set of mechanisms including molecular, cellular, neuroendocrine, physiological, and metabolic changes. These mechanisms are altered as a result of excess or lack of exposure to some nutrients, hormones, stress, and other conditions (e.g., placental dysfunction) during embryonic or fetal development. Hence, such factors especially during pregnancy characterize fetal programming and may encode the functions of organs or systems which may increase or decrease the risk for disease later in life (Institute of Medicine et al., 2009; Mennitti et al., 2018).

Maternal obesity has become a major health problem since it leads to many complications both in the mother and the fetus as well as metabolic disease risk programming of offspring later in life (Elshenawy and Simmons, 2016). Understanding how maternal obesity may affect the health outcomes of offspring has become a significant issue within the scientific world. Possible mechanisms that physiologically explain the link between maternal obesity and prenatal programming are epigenetic modifications such as DNA methylation, histone modification signatures, chromatin conformation, and microRNAs (non-coding RNAs) within some organs such as adipose tissue, liver, pancreas, and brain. These epigenetic alterations may cause the development of obesity and other related metabolic disorders in a developing fetus as well as in childhood and adulthood (Kitsiou-Tzeli and Tzetis, 2017; Montalvo-Martínez et al., 2018).

In recent years, the effects of perinatal nutrition (which is one of the environmental factors promoting prenatal programming) on the health of the fetus and the infant through the epigenome have been studied. It has been reported that maternal under- and overnutrition may permanently affect gene expression in epigenetic mechanisms of the fetus and these epigenetic modifications lead to metabolic abnormalities and also programming of obesity in prenatal life (Elshenawy and Simmons, 2016; Nicholas et al., 2016; Montalvo-Martínez et al., 2018). One of the main causes of the obesity epidemic is exposure to maternal undernutrition *in utero* which leads to intrauterine growth restriction (IUGR) in newborns. On the other hand, exposure to maternal obesity and maternal HFD *in utero* also lead to excessive weight gain in newborns. All these effects program the development of obesity beginning from the perinatal period as a result of the alterations in genes involved in many regulatory pathways such as energy metabolism, adipogenesis, glucose homeostasis, insulin signaling and hormone encoding like leptin and nuclear receptors (adipogenic and lipogenic transcription factors PPARγ and PPARα). (Alfaradhi and Ozanne, 2011; Desai and Ross, 2011; Desai et al., 2015; Elshenawy and Simmons, 2016).

It is demonstrated in the literature that the higher incidence of overweight and obesity in the offspring during the prenatal and neonatal period can be the result of the transmission of a greater number of susceptibility genes from obese and/or diabetic pregnant women to their offspring during pregnancy (Alfaradhi and Ozanne, 2011). Also in animal studies, the impact of maternal consumption of HFD during pregnancy on epigenetic changes in the liver and adipose tissue of the offspring through generations has been investigated. As a result, it has been indicated that maternal obesity which may be an adverse consequence of the consumption of maternal HFD is genetically transferred due to the transgenerational accumulation of many epigenetic modifications including histone methylation (Strakovsky et al., 2011; Li et al., 2012; Desai et al., 2015).

### Maternal excess weight gain and its short and long-term outcomes in offspring

The prevalence of maternal obesity and maternal excess weight gain has been gradually increasing worldwide (Valsamakis et al., 2015). Nutrient intake and weight gain during pregnancy are the two main modifiable risk factors influencing maternal and infant health outcomes (Institute of Medicine, 1990; Ota et al., 2011).

According to the Institute of Medicine (IOM) (2009) that are specific to pre-pregnancy body mass index (BMI), a gestational weight gain (GWG) of 12.5–18 kg for underweight women (BMI <18.5 kg/m^2^), 11.5–16 kg for normal weight women (BMI 18.5–24.9 kg/m^2^), 7–11.5 kg for overweight women (BMI 25.0–29.9 kg/m^2^) and 5–9 kg for obese women (BMI ≥30 kg/m^2^) are recommended (Institute of Medicine et al., 2009). Maternal obesity, which may be a consequence of excess weight gain during pregnancy, induces the development of pregnancy-related metabolic complications including gestational diabetes (GDM), pre-eclampsia, pregnancy-induced hypertension, maternal hemorrhage, miscarriage, preterm birth, and cesarean delivery. Also, the high prevalence of maternal adiposity and maternal obesity is linked with undesired perinatal side effects, such as fetal macrosomia (birth weight of >4,500 g), congenital abnormalities, stillbirth, etc. in the offspring (Korkmaz et al., 2016). Likewise, obese infants and children are at a greater risk of developing adulthood obesity and metabolic disorders including type 2 diabetes, cardiovascular disease, neurodevelopmental retardation, cancer, osteoporosis, and metabolic syndrome later in life. All of these mentioned health-related risk factors contribute to the maternal and fetal mortality rate (Valsamakis et al., 2015; Korkmaz et al., 2016; Catalano and Shankar, 2017; Goldstein et al., 2017; Harmon and Hannon, 2018; McDowell et al., 2019).

In a systematic review and meta-analysis of 45 studies, being overweight or obese during the pre-pregnancy period has been associated with an increased risk of high birth weight, macrosomia, and obesity in offspring (Yu et al., 2013). In a comprehensive study conducted by Stothard et al. (Stothard et al., 2009), it has been indicated that congenital malformations in children born to obese mothers are seen 2 times greater than the ones born to mothers with normal BMI. Meta-analysis of 12 cohort studies has reported that the risk of childhood overweight/obesity development is significantly increased as a result of maternal excessive weight gain in gestation (Tie et al., 2014). Likewise, another study conducted with 609 mother-child pairs who were followed until 36 months of *postpartum* showed that excessive GWG is associated with more than a 2-fold increase in the risk of childhood obesity (Diesel et al., 2015). In a cohort study by Reynolds et al. (Reynolds et al., 2019), the prevalence of obesity development in adolescent children born to obese mothers and mothers with GDM has been found as 40% and 26%, respectively. Therefore, according to the results of the comprehensive cohort studies, maintenance of maternal pre-pregnancy BMI, or simple dietary changes in addition to appropriate weight gain during pregnancy is a good strategy for preventing adverse pregnancy, neonatal and childhood outcomes (Guo et al., 2015; Maffeis and Morandi, 2017; Voerman et al., 2019).

### Potential mechanisms linking maternal overnutrition and maternal fatty acid intake to offspring obesity

Epidemiological studies investigate the metabolic effects of maternal factors on fetal body composition, neonatal birth weight, and childhood obesity (Frederick et al., 2008; Deierlein et al., 2011; Andres et al., 2012). One of the main reasons for the occurrence of maternal obesity and maternal excess weight gain is the consumption of the Western diet model that contains higher amounts of energy, sugar, and fat during pregnancy. Especially, the preference for high-fat and high-sugar diets in the gestational period leads to not only the development of maternal obesity but also the changes in the amount of some nutrients (e.g., glucose, fat, etc.) and metabolites that pass through the placenta, which both conditions can cause increase in fetal fat mass and fetal rapid weight gain (Muhlhausler and Ong, 2011; Ong et al., 2012). As a result of maternal overnutrition, it is shown that the increased placental transfer of nutrients to the developing fetus in obese mothers and mothers who gain excess weight during pregnancy, may subsequently affect fetal and neonatal body composition and metabolism; particularly including leptin expression increment in subcutaneous and visceral fat mass, leptin resistance and childhood obesity in postnatal life (Ay et al., 2009; Freeman, 2010; Muhlhausler and Ong, 2011; Ong et al., 2012; Kabaran, 2014; Montalvo-Martínez et al., 2018).

It has been stated that maternal obesity and excess GWG in pregnancy may result in permanent changes within the fetal neuroendocrine pathways in the hypothalamus, which control appetite regulation and energy metabolism in offspring (Poston et al., 2011). Eating behaviors are positively or negatively affected as a result of the changes in hypothalamic neural developmental pathways through altered insulin and leptin signaling. Therefore, these altered neuroendocrine factors may differ food preferences of offspring by causing an increment in fetal appetite and energy intake, consequently, all of these factors may contribute to alterations in the formation of body composition including fetal increased adiposity in offspring after birth (Muhlhausler et al., 2006; Lawlor et al., 2007; Brion et al., 2010; Reynolds et al., 2017). For instance, Kirk et al. (Kirk et al., 2009) have found an abnormal leptin surge in the rat neonates born to obese mothers in later life. It has also been stated in mice neonates that exposure to a maternal HFD leads to impaired hypothalamic neurocircuit formation due to the altered insulin signaling (Vogt et al., 2014). In studies conducted with animals (mostly with rodents, sheep, and non-human primates), neuronal changes in the hypothalamus were observed in the offspring of diabetic obese mothers caused by intrauterine exposures to metabolic factors. It has also been demonstrated that these metabolic factors *in utero* may increase the likelihood of developing diabetes and obesity in offspring (Korkmaz et al., 2016; Reynolds et al., 2017). Considering all these data, the accuracy of the hypothesis of *in utero* programming of obesity and metabolic syndrome has been proved.

Maternal obesity is also associated with adipose tissue inflammation, hyperlipidemia, and insulin resistance. In a systematic review and meta-analysis, it was found that maternal HFD is associated with higher body fat, body weight, leptin, glucose, insulin, and triglycerides levels, together with increased SBP in offspring in later life (Tellechea et al., 2017). In another systematic review and meta-analysis, it has been shown that excessive consumption of maternal HFD influences the development of visceral white adipose tissue in a murine offspring, related to adipocyte hypertrophy. In addition, hyperplasia was confirmed in the offspring in the long-term period (Saullo et al., 2022). Furthermore, maternal high-fat intake during pregnancy may lead to maternal peripheral tissue inflammation and insulin resistance which increase adipose tissue lipolysis, plasma FFA levels, and the activation of pro-inflammatory cytokines (McCurdy et al., 2009; Heerwagen et al., 2010). Increases in inflammation and plasma FFAs can modify the normal formation and developmental process of some organs in a fetus such as adipose tissue, pancreas, liver, brain, and skeletal muscle which increases the risk for metabolic disorders (Heerwagen et al., 2010).

Maternal dietary fatty acid intake, particularly the type and amount of dietary fatty acids are of great importance for the overall health of the mother and fetus (Aparicio et al., 2021). Among polyunsaturated fatty acids (PUFAs), linoleic acid 18:2 n-6 (LA) and alpha-linolenic acid 18:3 n-3 (ALA) are the two essential fatty acids. Both these essential fatty acids and their long-chain PUFAs, such as eicosapentaenoic (EPA), docosahexaenoic (DHA) and arachidonic (AA) acid play a crucial role in the fetal development (Aparicio et al., 2021; Duttaroy and Basak, 2022). In particular, n-3 fatty acids have anti-inflammatory effects, while n-6 fatty acids promote inflammation, highlighting the importance of the balance between n-3 and n-6 fatty acids (Duttaroy and Basak, 2022). It is suggested that high amounts of n-6 fatty acids in the pre-pregnancy and pregnancy period may adversely affect the fetal development and the overall health of offspring in later life (Aparicio et al., 2021).

For better metabolic outcomes both in the mother and the fetus, essential fatty acids and their long-chain PUFAs must be consumed in adequate and balanced amounts (Zhang et al., 2018). For instance, according to the Food and Agriculture Organization of the United Nations, a total of 300 mg per day of EPA and DHA, of which 200 mg per day DHA should be taken by pregnant and lactating women (Joint, 2010). The 2015–2020 Dietary Guidelines for Americans recommend that maternal diet in the perinatal period should consist of approximately 250 mg per day of EPA and DHA (US Department of Health and Human Services, 2015). In addition, it has also been shown that maternal supplementation with DHA up to 1 g per day or 2.7 g of EPA and DHA per day does not cause any adverse effects on maternal and infant health (Larqué et al., 2012). To benefit from these amounts of fatty acids, specifically DHA, it is recommended that pregnant or lactating women consume approximately 2 portions of a variety of seafood (especially oily fish) per week (US Department of Health and Human Services, 2015). Consequently, an optimal fatty acid profile in the mother’s diet is essential for maternal and fetal health in both the short and long-term (Aparicio et al., 2021). Exposure to excess fetal lipids in gestation may influence fetal growth and development, as well as contributing to the childhood obesity epidemic through fetal metabolic programming (McCurdy et al., 2009; Heerwagen et al., 2010).

### The Impact of Maternal HFDs on Offspring Obesity by Altering the Epigenetic Mechanisms of Hypothalamic Appetite and Reward Systems

**FIGURE 1:**
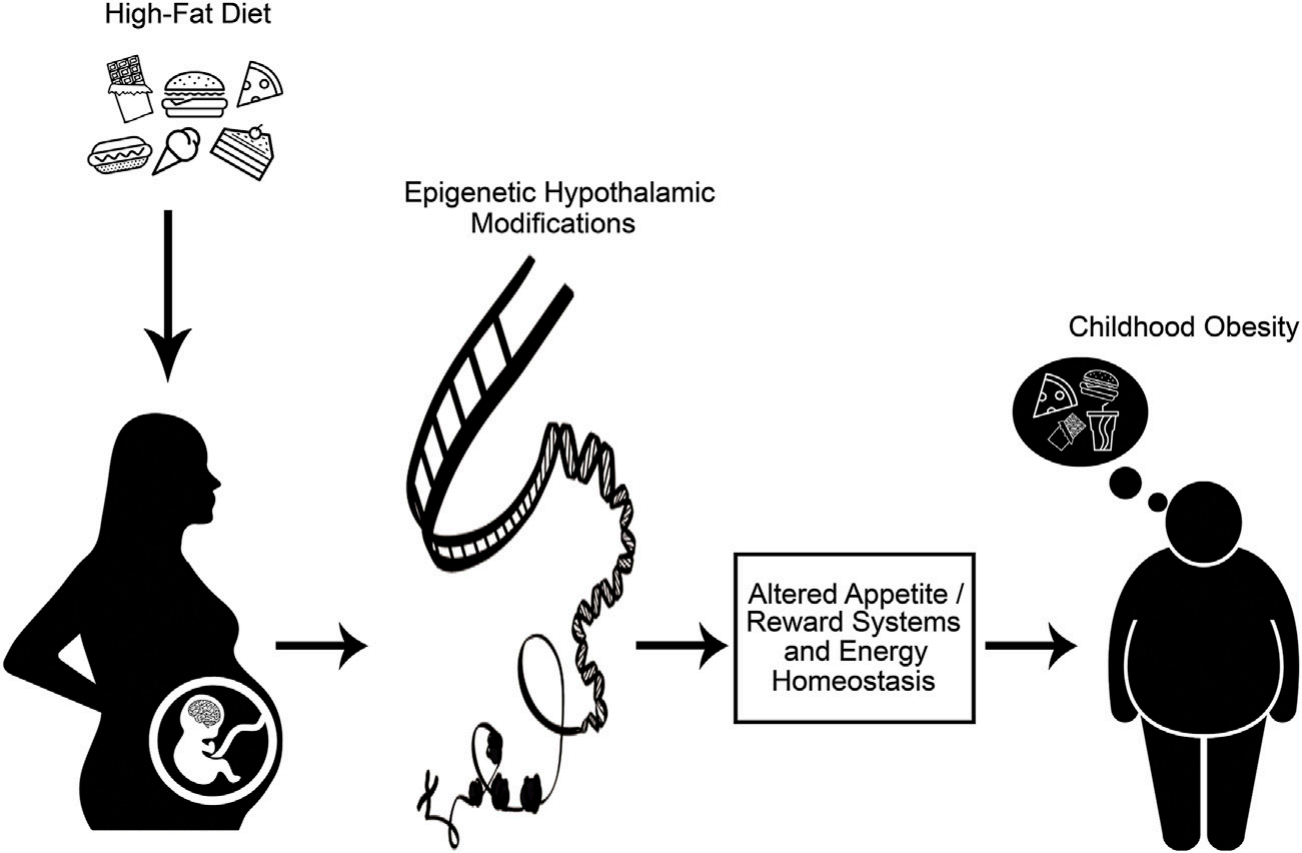
Maternal consumption of high-fat diet alters epigenetic programming of appetite, reward systems and energy homeostasis in fetus, leading to development of childhood obesity.

Evidence from clinical and experimental studies have investigated the relationship between maternal obesogenic environment, gene-environment interactions, and developmental programming of obesity. Data from those studies have also explained the occurrence of obesity-related health outcomes in offspring through epigenetic modifications (Ota et al., 2011; Reddon et al., 2016; Hsu and Tain, 2019). Maternal nutrition, which is one of the main environmental factors that interact with genes, and also many other perinatal exposures, play a critical role in the regulation of the hypothalamic neuroendocrine pathways that control appetite and energy homeostasis in offspring (Levin, 2008). Thus, explanation of the influences of maternal overnutrition (particularly overconsumption of HFD) and maternal obesity through epigenetic alterations on such neuroendocrine mechanisms involved in energy homeostasis have gained huge importance for understanding the alterations in appetite and reward systems of offspring, and also the treatment and prevention of obesity (Figure 1). (Chen et al., 2009; Morris, 2009)

The regulation of fetal appetite and energy homeostasis in the hypothalamus starts in the perinatal period, and hence, specific alterations in the hypothalamic appetite and energy regulatory pathways can directly influence the appetite and also food intake during critical periods of life (Ross and Desai, 2014). Hypothalamic appetite regulatory site of the hypothalamus, the arcuate nucleus (ARC), involves appetite-stimulated orexigenic neurons such as NPY and AgRP, and also appetite-suppressor anorexigenic neurons such as POMC and cocaine- and amphetamine regulating transcript (CART). These neurons are responsible for the regulation of nutrition through central and peripheral signals (Ross and Desai, 2013; Ross and Desai, 2014). Therefore, maternal nutrition and maternal obesity during the fetal and/or postnatal period may permanently program ARC structure and function, and influence appetite (Morris, 2009).

Studies strongly indicate that mechanisms that program fetal appetite in pregnancy are affected by maternal nutrition (undernutrition and/or overnutrition) consequently increasing the susceptibility to hyperphagia and the risk of later obesity in offspring. Of note, it has been reported that these perinatally programmed appetite mechanisms involve multiple factors/pathways such as altered hypothalamic ARC neuropeptides, neuroendocrine signaling and/or epigenetics by exposure to maternal HFD (Ross and Desai, 2014). It has been showing in animal models, mostly in rodents that infants born as a small for gestational age (SGA) as a consequence of maternal undernutrition exhibit a rapid catch-up growth with hyperphagia by showing impaired satiety cell signaling and enhanced cellular orexigenic responses (Guo et al., 2002; Desai et al., 2005). Furthermore, in a study with infants born to obese mothers who consumed a HFD in gestation, infants had hyperphagia and therefore rapid neonatal growth (Vickers et al., 2000). Both conditions during pregnancy lead to enhanced orexigenic neuropeptide responses. Therefore, adulthood obesity is increased as a result of pregnancy-programmed hyperphagia (Vickers et al., 2000; Taylor and Poston, 2007; Chen et al., 2009).

Leptin and insulin hormones, which are secreted by adipose tissue and pancreas organs respectively, are anorexigenic factors that inhibit the NPY/AgRP neurons and activate the POMC neurons. These anorexigenic hormones provide a negative feedback mechanism in the hypothalamus to inhibit food intake. However, both hormones are over-secreted in obese people, and they perform a resistance in peripheral and central tissues which leads to the development of metabolic disorders (Cesar and Pisani, 2017). Beside their critical role in regulating energy homeostasis, leptin and insulin are also critical mediators of neural development during the pre- and postnatal periods.^567^ Maternal HFD intake during both pregnancy and lactation as well as a maternal food restriction result in impaired leptin, and insulin sensitivity and appetite/satiety gene expression in offspring. For example, it has been found in animals that maternal obesity and maternal consumption of HFD in lactation increases hypothalamic expression (mRNA and protein) of NPY and AgRP, whereas reduces POMC expression (Chen et al., 2009; Stofkova et al., 2009; Desai et al., 2016) and decreases sensitivity to leptin (Patterson et al., 2008). Also, maternal HFD intake during gestation has caused neonatal impaired hypophagic response to insulin as adults (Sardinha et al., 2006). Additionally, it has been demonstrated that the NPY and POMC neurons are being modified by chronic overconsumption of HFD, and are associated with the development of metabolic disorders, such as increased adiposity, hyperinsulinemia, and insulin resistance. It has been reported that perinatal hyperinsulinemia induces resistance against the regulatory signals of the ARC system which are leptin and insulin, leading to hyperphagia and overweight in older offspring (Chen et al., 2009; Galjaard et al., 2013).

Pre-pregnancy and pregnancy are critical periods in terms of proper programming of epigenetic mechanisms. For this reason, exposure to various environmental, and nutritional factors in those critical periods may influence DNA methylation throughout adult life (Martin and Fry, 2018). It has been stated that maternal nutrition, maternal body composition and other endocrine factors affect some important gene methylations in fetal energy metabolism and these methylations are mainly responsible for the epigenetic changes in DNA methylation (Vucetic et al., 2010; Borengasser et al., 2013). Also, both human and animal studies have reported that DNA methylation, which occurs primarily at cytosine phosphate-guanine (CpG) dinucleotides site, may modulate POMC expression. Hence, maternal nutrition in pre- and postnatal periods may alter appetite and obesity-related phenotype by influencing POMC gene methylation, and this may be transferred to childhood and adulthood (Reynolds et al., 2017; Candler et al., 2019). For instance, Ramamoorthy et al. (Ramamoorthy et al., 2018) have found in their experimental study that maternal HFD programs long-term epigenetic alterations in the hypothalamic POMC gene of offspring by causing alterations in DNA methylation. Likewise, Plagemann et al. (Plagemann et al., 2009) have demonstrated in a rodent model that POMC hypermethylation, as a result of exposure to a dietary high-energy in the neonatal period, may suppress the satiety response by inhibiting the action of leptin and insulin signaling. There are also evidence showing that consumption of high-energy diets (especially the Western diet model) during the postnatal period increases the expression of both POMC and AgRP/NPY neurons by inducing epigenetic modifications, leading overall to overnutrition and increased body weight (Lazzarino et al., 2017). It has also been reported in a study that AgRP/NPY genes are more susceptible to postnatal overnutrition than POMC, and may be more implicated with the postnatal phenotype (Lazzarino et al., 2017).

Although there are many factors contributing to the infant, childhood and, adulthood obesity epidemic, the association between the exposure to Western diet model during gestation and/or early infancy and an increased preference for high-fat, high-sugar foods (palatable/junk foods) in the offspring during postnatal life is still been investigating (Bayol et al., 2008; Ong et al., 2012). One of the possible mechanisms for the programming of palatable food preferences in offspring is the mesolimbic reward system in the brain which includes the nucleus accumbens (NAc) and ventral tegmental area (VTA). The key systems involved in mediating this effect are the dopaminergic and opioid signaling systems within the mesolimbic reward system (Ong et al., 2012; Grissom et al., 2014). Thereby, molecules (dopamine and opioids) that participate in regulating the consumption of palatable foods in these brain areas have been indicated to be altered in offspring born from obese mothers or mothers fed a HFDs which may result in changes in eating behavior of offspring (Johnson and Kenny, 2010; Vucetic et al., 2010; Sullivan et al., 2015; Reynolds et al., 2017). The main mechanism of this result is the changes in DNA methylation and gene expression of dopamine and opioid-related genes (including the dopamine reuptake transporter; DAT and the µ-opioid receptor; MOR) by exposure to maternal HFD during the prenatal and postnatal periods (Reyes, 2012; Grissom et al., 2014). Furthermore, besides DNA methylation, histone modifications can also be modulated by nutritional factors. These modifications can influence eating behavior and maintenance of body weight (Aagaard-Tillery et al., 2008; Funato et al., 2011; Şanlı and Kabaran, 2019). In animal studies, it has been reported that exposure to maternal high-fat/high-sugar diets has been shown to result in altered expression of genes in central reward systems, leading to an increase in fat intake in offspring in later life (Ong and Muhlhausler, 2011). Likewise, Rivera et al. (Rivera et al., 2015) have revealed that non-human offspring primates exposed to both maternal HFD and maternal obesity during early development are at increased risk for obesity, as a result of altered central dopamine signaling. Also, Vucetic et al. (Vucetic et al., 2010) have shown significant changes occur in both opioid and dopamine systems in response to exposure to a maternal HFD during pregnancy and lactation. Apart from the mentioned studies, some contrasting findings have been reported about the effects of maternal HFD on the offspring’s food preferences and obesity development during the prenatal and/or postnatal periods. For example, it is demonstrated that exposure to a maternal HFD *in utero* plays an important role in programming food preferences by altering the expressions of some neurons in central the reward system (Chang et al., 2008), whereas in another study, it has been shown that exposure to a maternal HFD in lactation period is more sensitive for programming food preferences and development of obesity in later life (Bayol et al., 2007). Thus, further investigations into the issue will explain better the mechanisms and critical windows for the programming of food preferences and obesity.

The inflammation associated with obesity has been shown to occur not only in peripheral tissues (e.g., adipose tissue, liver, pancreas, etc.), but also in the central nervous system. Recently, a strong association has been found between maternal consumption of HFD, hypothalamic inflammation, and the disruption on hypothalamic appetite and energy metabolism control in offspring (Valdearcos et al., 2014; Jais and Brüning, 2017; Le Thuc et al., 2017). This disruption at an early stage of development could set the susceptibility to develop obesity and non-communicable diseases in later life. It has been shown that the inflammatory response to both acute and chronic consumption of HFD (especially excess levels of saturated fatty acids; SFAs, and omega-6 PUFAs) is mediated by Toll-like receptor (TLR) signaling, activation of nuclear factor κ-B (NF-κB) and production of proinflammatory cytokines such as tumor necrosis factor (TNF)-α, interleukin (IL)-1β and IL-6. Also, TNF-α has been reported to modulate hypothalamic neuropeptides involved in appetite regulation (Dalvi et al., 2017; Le Thuc et al., 2017; Poon, 2020). In addition, as previously mentioned, hypothalamic leptin and insulin resistance, induced by hypothalamic inflammation as a result of HFD, also lead to alterations in the homeostatic regulation of hunger and satiety (De Souza et al., 2005; Enriori et al., 2007).

The hypothalamic inflammation induced by exposure to maternal HFD has been confirmed in animals. The overexpression of inflammatory markers within neurons is seen in response to maternal HFD feeding (especially prolonged high-fat feeding). This mechanism leads to an alteration of the expression of certain hypothalamic neuropeptides and occurs a positive energy balance in offspring (Le Thuc et al., 2017). For instance, Dalvi et al. (Dalvi et al., 2017) reported that chronic inflammation as a result of chronic HFD feeding causes an increase in TNF-α expression in the NPY/AgRP neurons, favoring an increase in appetite and neuropeptide dysregulation inclined toward energy intake. Likewise, Shi et al. (Shi et al., 2013) demonstrated that HFD-induced chronic inflammation inhibited the activation of POMC transcription of in male mice In a study carried out on non-human primates by Grayson et al. (Grayson et al., 2010), it has been found alterations in both POMC mRNA and AgRP mRNA by activation of proinflammatory cytokines in the fetal hypothalamus as a result of prolonged maternal exposure an HFD. Consequently, studies have reported that especially an HFD, even in the short term, can induce inflammation in the hypothalamus and that would play a major role in the pathophysiology of obesity (Le Thuc et al., 2017).

## Conclusion

Obesity involves the complex interaction of various environmental and genetic factors. Alterations in the metabolic environment during critical periods of fetal development can predispose individuals to the later development of obesity. Considering all these effects, fetal programming of obesity and obesity-related diseases has been a growing target of interest in the scientific world, especially in terms of nutritional perspective. Unfavorable maternal nutrition and/or other several environmental factors lead to permanent changes in fetal metabolic and epigenetic mechanisms. It has been widely discussed in studies that increased consumption of maternal dietary fats during pregnancy has contributed to an increase in fetal fat mass, fetal rapid weight gain, and the programming of obesity. Hence, as a result of exposure to excess maternal dietary fats and maternal obesity, alterations in the epigenetic mechanisms of appetite and reward systems and energy homeostasis occur, which lead to changes in eating behavior and consequently the development of obesity in later life. Furthermore, detailed guidance by the healthcare providers, specifically by the dietitians on the types and amounts of foods, nutrients, and dietary patterns during the perinatal period may be the potential interventions for the improvement of maternal and fetal metabolic health. Moreover, providing dietetic counseling services that specifically evaluate dietary fat and fatty acid intake in the perinatal period, as well as nutrition education on the effects of dietary fatty acid types and fatty acid intake on maternal and fetal health may increase awareness and enable mothers to choose healthy foods. Consequently, maternal dietary interventions are the main adjustable factor for improving the maternal and fetal metabolic environment. The effects of maternal dietary fat and fatty acid intake on maternal and fetal health, as well as offspring obesity should be further investigated and evaluated with randomized controlled trials.
